# Implementation of Continuous Capnography Is Associated With a Decreased Utilization of Blood Gases

**DOI:** 10.14740/jocmr1997w

**Published:** 2014-11-19

**Authors:** Courtney M. Rowan, Richard H. Speicher, Terri Hedlund, Sheikh S. Ahmed, Nancy L. Swigonski

**Affiliations:** a Department of Pediatrics, Indiana University School of Medicine, Indianapolis, IN, USA; b Department of Pediatrics, University Hospitals of Cleveland, Cleveland, OH, USA; c Pediatric Critical Care Nursing, Indiana University Health, Riley Hospital for Children, Indianapolis, IN, USA; d Fairbanks School of Public Health, Indiana University Purdue University, Indianapolis, IN, USA

**Keywords:** Mechanical ventilation, Critical care, Carbon dioxide, Quality improvement

## Abstract

**Background:**

Capnography provides a continuous, non-invasive monitoring of the CO_2_ to assess adequacy of ventilation and provide added safety features in mechanically ventilated patients by allowing for quick identification of unplanned extubation. These monitors may allow for decreased utilization of blood gases. The objective was to determine if implementation of continuous capnography monitoring decreases the utilization of blood gases resulting in decreased charges.

**Methods:**

This is a retrospective review of a quality improvement project that compares the utilization of blood gases before and after the implementation of standard continuous capnography. The time period of April 2010 to September 2010 was compared to April 2011 to September 2011. Parameters collected included total number of blood gases analyzed, cost of blood gas analysis, ventilator and patient days.

**Results:**

The total number of blood gases after the institution of end tidal CO_2_ monitoring decreased from 12,937 in 2009 and 13,171 in 2010 to 8,070 in 2011. The average number of blood gases per encounter decreased from 20.8 in 2009 and 21.6 in 2010 to 13.8 post intervention. The blood gases per ventilator day decreased from 4.94 in 2009 and 4.76 in 2010 to 3.30 post intervention. The total charge savings over a 6-month period was $880,496.

**Conclusions:**

Continuous capnography resulted in a significant savings over a 6-month period by decreasing the utilization of blood gas measurements.

## Introduction

Capnography provides a continuous, non-invasive monitoring of the carbon dioxide (CO_2_) in respiratory gases to assess adequacy of ventilation. These monitors, in addition to providing a continuous measurement of CO_2_, provide added safety features in mechanically ventilated patients by allowing for quick identification of unplanned extubation. A sudden loss of the capnography tracing allows the physician to quickly be alerted to a potential disaster, such as endotracheal tube dislodgement or obstruction [1]. A significant, sudden decrease in the end tidal carbon dioxide (ETCO_2_) reading can also indicate deficiency of pulmonary blood flow, such as with a cardiac arrest or pulmonary embolism [1]. It has been shown to be helpful in cardiopulmonary resuscitation as a sign of return of spontaneous circulation [2].

Beyond its use as a safety measure, capnography may also be useful for ventilator weaning [1, 3-5]. It correlates well with the PaCO_2_ obtained from arterial blood gas analysis [4], with a difference usually less than 5 mm Hg in normal physiology [6]. Changes made to the ventilator can be quickly assessed with the display of a continuous CO_2_ level on the monitor. While one study found no change in the amount of blood gases analyzed [7], another has found a reduction [4].

Adult data demonstrate compelling safety reasons for continuous capnography to be routinely implemented in mechanically ventilated patients [1, 2]. A group of pediatric intensivists at our institution championed the implementation of standardized continuous side stream capnography to address patient safety. Specifically, we hypothesized that in addition to the known safety advantages, these monitors would allow for decreased utilization of blood gases and there would be a resultant decrease in charges.

## Methods

### Subjects, setting and procedures

This study was approved by our institutional review board (IRB #1108006536) prior to any data collection or analysis. Our initial purchase of the ETCO_2_ monitors was approximately $111,700. In March 2011, standard continuous side stream capnography was implemented for all mechanically ventilated patients in the pediatric intensive care unit (PICU). All intubated patients were required to have ETCO_2_ monitoring after the implementation. Patients from the cardiac critical care service received ETCO_2_ monitoring but were excluded from this study prior to any analysis since blood gases were frequently obtained when assessing electrolytes, acid/base balance, and lactate trends.

Discussions regarding the standardization of continuous capnography began 6 months prior to implementation involving the section of pediatric critical care, nursing, and respiratory therapy leadership. Approximately 3 months prior to implementation, general educational sessions were held. These sessions took place via email, at the PICU faculty meeting, and education sessions with nursing staff. The respiratory therapists (RTs) received the most substantial education and training. In the month leading up to the employment of the monitors, more intensive in-service sessions were held with RTs. As with the implementation of any new technology, there was resistance from a few. This was addressed with additional education of physicians, RTs, and nurses. These sessions consisted of descriptions of ETCO_2_ monitor, how it could be utilized, and emphasized why it may benefit our patients’ safety.

The section of respiratory therapy was charged with the task to ensure that all intubated and mechanically ventilated patients had ETCO_2_ monitoring. Multiple educational sessions were conducted with respiratory therapy on how to connect and set up the monitors. These monitors were then made to become part of their standard ventilator check list. There was also the expectation of the RT to document the ETCO_2_ along with all other ventilator and respiratory parameters. An additional fail-safe was that the order to perform continuous capnography was incorporated into our standard ventilation order set.

Weaning of the ventilator and orders for blood gas analysis were at the discretion of the pediatric intensivist in charge of the patient’s care. There were no other significant changes to the care of the mechanically ventilated patient during the study period. Nursing staff, physicians, and RTs involved in the routine care of the patients were unaware of the study.

### Measures

An observational study was then conducted to analyze the utilization of blood gas measurement and resultant changes in charges in the PICU. All blood gases obtained were point of care (POC) analysis, i.e. done at the bedside by the critical care nurse. We retrospectively analyzed the time period of April-September 2009 and April-September 2010 and compared those two time periods to April-September 2011 in order to monitor trends before and after initiation of capnography. Parameters collected included total number of blood gases analyzed, charges of blood gas analysis, ventilator days, and patient days. The data were collected from clinical decision support and hospital accounting.

Descriptive statistics using means and standard deviations were calculated for the total number of blood gases, the total charges of blood gases, the number of patients admitted to the PICU per month requiring at least one blood gas analysis, the number of blood gases per individual, number of ventilator days per month, number of blood gases per ventilator day and total blood gas charges, and blood gas charges per patient and per ventilator day. Comparisons of our two main outcomes (number of blood gases and total charges) were compared using ANOVA with Tukey *post hoc* analysis for the three time periods April-September 2009, April-September 2010 and April-September 2011 (the same months as the timeframe for our intervention). We used Statistical Package of the Social Science (SPSS) Statistical software for Windows, Version 20.0 (SPSS Inc., Chicago, IL, USA) and Microsoft Office Excel (Microsoft Corporation, Redmond, WA, USA).

## Results

There was a statistically significant decrease in the total number of blood gases analyzed after the institution of continuous ETCO_2_ monitoring compared to the same time period from the prior years (Table 1). A comparison of the number of patients admitted to the PICU per month requiring at least one gas analysis was not significantly different before versus after the initiation of standard ETCO_2_ monitoring blood (103.8 ± 17.8 and 101.5 ± 6.4 vs. 96.8 ± 5.6, P = 0.57). There was also a significant decrease in the average number of blood gases analyzed per patient.

**Table 1:** Summary of Changes in Blood Gas Analysis Over a 3-Year Period

	April-September 2009	April-September 2010	April-September 2011	P value
Total number of blood gases analyzed	12,937	13,171	8,070	0.001
Average number of blood gases per patient encounter	20.8 (3.8)	21.6 (1.4)	13.8 (1.7)	< 0.0001
Average number of blood gases per ventilator day	4.94 (0.79)	4.76 (0.75)	3.30 (0.79)	0.004

P values obtained from a one-way ANOVA.

**Figure 1:**
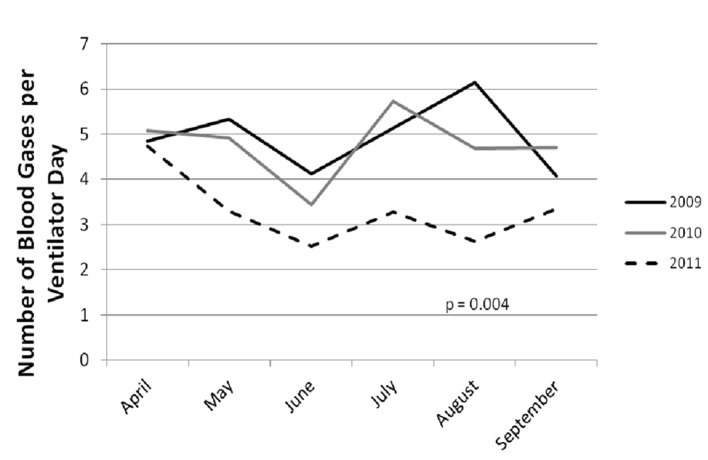
Mean blood gases per day are displayed for each month of the years 2009, 2010, and 2011. It is noted that the average blood gas utilization per day is consistently lower in each month of 2011 than in the preceding 2 years.

The average number of ventilator days per month was not significantly different (431.3 ± 57.7 and 474.0 ± 99.0 vs. 415.2 ± 56.4, P = 0.38). There was a significant decrease in the average number of blood gases analyzed per ventilator day (Table 1, Fig. 1).

We examined the mean blood gas charges per patient encounter and mean blood gas charges per ventilator day. We found a decrease in both of these charges (Table 2). The total blood gas charge decreased from $2,207,804 in 2009, $2,261,051 in the study time period in 2010 to $1,544,360 in 2011. Comparing the study time period in 2010 to 2011, there was a total savings of $716,691 (Table 2). During this time period, however, the charges for an individual blood gas analysis increased. Using the charge for a blood gas in 2009 to calculate total charges, the savings rises to nearly $1 million ($880,496). Our initial purchase of the ETCO_2_ monitors was approximately $111,700, therefore the total savings over a 6-month period was $768,796.

**Table 2:** Summary of Changes in Blood Gas Charges Over a 3-Year Period (Mean, Standard Deviation (Median))

	Charges April-September				Charges in 2010 - 2009		P-value	
	2009	2010	2011 actual*	2011 converted^#^	Actual savings	Converted savings	Actual	Converted
Total charge of blood gases analyzed	$2,207,804	$2,261,051	$1,544,360	$1,380, 556	$716,691	$880,495	0.02	0.005
Charge of blood gas analysis per ventilator day	$839 ± 172 ($830)	$817 ± 129 ($825)	$631 ± 150 ($631)	$564 ± 134 ($564)	$186	$253	0.06	0.009
Charge of blood gas analysis per patient encounter	$3,522 ± 241 ($3,242)	$3,716 ± 241 ($3,695)	$2,649 ± 328 ($2,589)	$2,368 ± 293 ($2,313)	$1,067	$1,348	0.007	0.001

Values are means ± standard deviation. Medians are displayed in parenthesis. P values obtained from a one-way ANOVA. *2011 actual are the actual charges for blood gases during the study time period April-September. #2011 converted are the charges re-calculated using the lower 2010 charge per blood gas, i.e. the charge, if there had not been an increase in the price of a blood gas between 2010 and 2011.

## Discussion

Our study found a dramatic, almost 40%, decrease in the number of blood gases analyzed since the institution of standard continuous capnography over a 6-month period. This drop in blood gas utilization is most likely attributed to the ETCO_2_ monitoring. Although we did not have a measure of patient acuity, over the time periods that were compared there were a similar number of patient encounters with blood gas measurements and a similar number of ventilator days. Comparing the number of blood gases per ventilator day takes into account some of the patient acuity and ensures that we did not have fewer blood gases because we had fewer children requiring mechanical ventilation. The average number of blood gases analyzed per patient day and per ventilator day was statistically significant, strengthening the conclusion that there is a genuine decrease in blood gas usage.

The implementation was championed and well supported from a physician standpoint because most agreed that it improved patient safety by having an additional method to alert the care provider to unplanned extubation. Prior to that time, main stream capnography was available, but not routinely utilized because of an overwhelming barrier, i.e. the weight of the main stream capnographer caused kinking in the endotracheal tube, particularly smaller caliber tubes. This concern was addressed by using side stream capnography, which does not directly attach to the endotracheal tube.

One limitation to this study is that we are unable to determine retrospectively why a blood gas was ordered. In our PICU, POC analyses may be ordered to assess metabolic or electrolyte disturbances. The cardiac critical care patients were excluded from this study, as these are the patients that most frequently have POC blood gases analyzed for reasons other than assessment of the respiratory status. However, we would not expect a significant change in the number of blood gases analyzed for metabolic or electrolyte reasons and if we were able to exclude these types of blood gases, we may have found an even greater decrease in those used for strictly respiratory reasons.

One may speculate that decrease blood gases may lead to other areas of improvement for quality, safety, and costs. While these other implications would obviously need to be studied specifically, it is interesting to reflect on the effects that decreased blood gas analysis may have. For example, by decreasing the number of blood gases analyzed, there is a concurrent decrease in the amount of times the central line or arterial line is accessed. Frequent blood draws from invasive catheters have been shown to increase the risk of catheter associated blood stream infections (CABSIs) [8, 9]. The cost of a CABSI has been estimated to be anywhere from US$21,400 to US$110,800 [10]. CABSIs have also been shown to have the highest mortality of any hospital acquired infection [10]. If continuous capnography can reduce the risk of CABSI by decreasing the number of times an invasive line is accessed, then it may result in a reduced mortality rate not related to the identification of ventilation problem. The 40% reduction in blood gas analysis has certainly led to a significant reduction in the number of times a line is accessed, potentially decreasing the risk of catheter-related infections. CABSI rates have fallen at our institution over the 3 years of the study period but there have been other quality improvement efforts specifically targeted to CABSI, so attribution to capnography is difficult.

As another example, by reducing the number of blood draws, there may also be a reduction in iatrogenic anemia. While the amount of blood required for the actual POC analysis is small, there can be a significant amount of blood loss when drawing from a line that requires a few milliliters of waste to reduce contamination of the gas results. A few milliliters of blood several times a day may cause iatrogenic anemia, especially in small critically ill patients. This may lead to a small reduction in the number of blood transfusions required. The cost of a blood transfusion can be anywhere from $522 to $1,183 per unit [11], which can increase the amount of savings seen from the implementation of continuous capnography.

### Conclusion

Continuous capnography can not only improve patient safety and allow for a non-invasive means of ventilator weaning, but also result in a significant savings by decreasing the utilization of blood gas measurements. At our institution we were successful at implementing a quality improvement project around capnography monitoring that may have improved patient safety and led to a total savings of $768,796 over a 6-month period.
